# Optimizing Provider Recruitment for Influenza Surveillance Networks

**DOI:** 10.1371/journal.pcbi.1002472

**Published:** 2012-04-12

**Authors:** Samuel V. Scarpino, Nedialko B. Dimitrov, Lauren Ancel Meyers

**Affiliations:** 1 The University of Texas at Austin, Section of Integrative Biology, Austin, Texas, United States of America; 2 Naval Postgraduate School, Operations Research Department, Monterey, California, United States of America; 3 The Santa Fe Institute, Santa Fe, New Mexico, United States of America; University of New South Wales, Australia

## Abstract

The increasingly complex and rapid transmission dynamics of many infectious diseases necessitates the use of new, more advanced methods for surveillance, early detection, and decision-making. Here, we demonstrate that a new method for optimizing surveillance networks can improve the quality of epidemiological information produced by typical provider-based networks. Using past surveillance and Internet search data, it determines the precise locations where providers should be enrolled. When applied to redesigning the provider-based, influenza-like-illness surveillance network (ILINet) for the state of Texas, the method identifies networks that are expected to significantly outperform the existing network with far fewer providers. This optimized network avoids informational redundancies and is thereby more effective than networks designed by conventional methods and a recently published algorithm based on maximizing population coverage. We show further that Google Flu Trends data, when incorporated into a network as a virtual provider, can enhance but not replace traditional surveillance methods.

## Author Summary

Public health agencies use surveillance systems to detect and monitor chronic and infectious diseases. These systems often rely on data sources that are chosen based on loose guidelines or out of convenience. In this paper, we introduce a new, data-driven method for designing and improving surveillance systems. Our approach is a geographic optimization of data sources designed to achieve specific surveillance goals. We tested our method by re-designing Texas' provider-based influenza surveillance system (ILINet). The resulting networks better predicted influenza associated hospitalizations and contained fewer providers than the existing ILINet. Furthermore, our study demonstrates that the integration of Internet source data, like Google Flu Trends, into surveillance systems can enhance traditional, provider-based networks.

## Introduction

Since the Spanish Flu Pandemic of 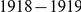, the global public health community has made great strides towards the effective surveillance of infectious diseases. However, modern travel patterns, heterogeneity in human population densities, proximity to wildlife populations, and variable immunity interact to drive increasingly complex patterns of disease transmission and emergence. As a result, there is an increasing need for effective, evidence-based surveillance, early detection, and decision-making methods [1]–[3]. This need was clearly articulated in 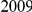 by a directive from the Department of Homeland Security and the Centers for Disease Control and Prevention to develop a nationwide, real-time public health surveillance network [4], [5].

The U.S. Outpatient Influenza-Like Illness Surveillance Network (ILINet) gathers data from thousands of healthcare providers across all fifty states. Throughout influenza season (CDC mandating reporting during weeks 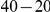, which is approximately October through mid-May), participating providers are asked to report weekly the number of cases of influenza-like illness treated and total number of patients seen, by age group. Cases qualify as ILI if they manifest fever in excess of 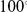
F along with a cough and/or a sore throat, without another known cause. Although the CDC receives reports of approximately 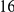 million patient visits per year, many of the reports may use a loose application of the ILI case definition and/or may simply be inaccurate. The data are used in conjunction with other sources of laboratory, hospitalization and mortality data to monitor regional and national influenza activity and associated mortality. Similar national surveillance networks are in place in 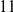 EU countries and elsewhere around the globe [6]–[9].

Each US state is responsible for recruiting and managing ILINet providers. The CDC advises states to recruit one regularly reporting sentinel provider per 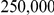 residents, with a state-wide minimum of 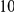 sentinel providers. Since 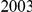, the Texas Department of State Health Services (DSHS) has enrolled a total of 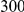 volunteer providers. Participating providers regularly drop out of the network; Texas DSHS aims to maintain approximately 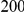 active participants through year-round recruitment of providers in heavily populated areas (cities with populations of at least 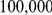). DSHS also permits other (non-targeted) providers of family medicine, internal medicine, pediatrics, university student health services, emergency medicine, infectious disease, OB/GYN and urgent care to participate in the network. During the 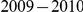 influenza season, the Texas ILINet included 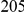 providers with approximately 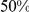 reporting most weeks of the influenza season.

A number of statistical studies have demonstrated that ILI surveillance data is adequate for characterizing past influenza epidemics, monitoring populations for abnormal influenza activity, and forecasting the onsets and peaks of local influenza epidemics [10]–[16]. However, the surveillance networks are often limited by non-representative samples [17], inaccurate and variable reporting [12]–[14], and low reporting rates [6]. Some of these studies have yielded specific recommendations for improving the performance of the surveillance network, for example, inclusion of particular categories of hospitals in China [12], preference for general practitioners over pediatricians in Paris, France [14], and a general guideline to target practices with high reporting rates and high numbers of patient visits (per capita) [6]. Polgreen et al. 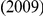 recently described a computational method for selecting ILINet providers so as to maximize coverage, that is, the number of people living within a specified distance of a provider [17]. They applied the approach to optimizing the placement of the 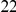 providers in the Iowa ILINet. While their algorithm ensures maximum coverage, it is not clear that maximum coverage is, in general, the most appropriate criterion for building a statistically informative ILINet.

In 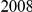, Google.org launched Google Flu Trends, a website that translates the daily number of Googles search terms associated with signs, symptoms, and treatment for acute respiratory infections into an estimate of the number of ILI patients per 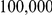 people. It was shown that Google Flu Trends reliably estimates national influenza activity in the US [18], the state of Utah [18], and in some European countries [19], but it provided imperfect data regarding the 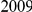 H1N1 pandemic in New Zealand [20]. We assessed the correlation between Google Flu Trends for Texas and Texas' ILINet data and found a correlation of 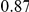, similar to those presented in Ginsberg et al. 2009 [18] (See Text S1). The Google Flu Trends website includes ILI-related search activity down to the level of cities (in beta version as of November 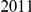). Thus, Google Flu Trends may serve as a valuable resource for influenza detection and forecasting if effectively integrated with public health data such as those coming from state ILINets.

Here, we present an evaluation of the Texas Influenza-Like-Illness Surveillance Network (ILINet), in terms of its ability to forecast statewide hospitalizations due to influenza (ICD9 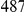 and 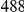) and unspecified pneumonia (ICD9 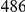). Although we henceforth refer to this subset of hospitalizations as *influenza-like hospitalizations*, we emphasize that these data do not perfectly reflect influenza-related hospitalizations: some unrelated pneumonias may be classified under ICD9 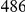, and some influenza cases may not be correctly diagnosed and/or recorded as influenza. Nonetheless, this subset of hospitalizations likely includes a large fraction of hospitalized influenza cases and exhibits strong seasonal dynamics that mirror ILINet trends. The inclusion of all three ICD9 codes was suggested by health officials at Texas DSHS who seek to use ILINet to ascertain seasonal influenza-related hospitalization rates throughout the state (Texas DSHS contract numbers 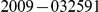 and 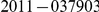). Hospitalizations associated with these three codes in Texas accounted for between 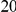 and 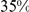 of all hospitalizations due to infections and roughly 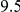 billion dollars of hospitalization payments in 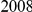 (See Text S1).

Using almost a decade of state-level ILINet and hospitalization data, we find that the existing network performs reasonably well in its ability to predict *influenza-like hospitalizations*. However, smaller, more carefully chosen sets of providers should yield higher quality surveillance data, which can be further enhanced with the integration of state-level Google Flu Trends data. For this analysis, we adapted a new, computationally tractable, multilinear regression approach to solving complex subset selection problems. The details of this method are presented below and can be tailored to meet a broad range of surveillance objectives.

## Results

Using a submodular ILINet optimization algorithm, we investigate two scenarios for improving the Texas ILINet: designing a network from scratch and augmenting the existing network. We then evaluate the utility of incorporating Google Flu Trends as a virtual provider into an existing ILINet.

### Designing a New ILINet

To construct new sentinel surveillance networks, we choose individual providers sequentially from a pool of approximately 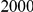 mock providers, one for each zip code in Texas, until we reach 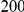 total providers. At each step, the provider that most improves the quality of the epidemiological information produced by the network is added to the network. We optimize and evaluate the networks in terms of the time-lagged statistical correlation between aggregated ILINet provider reports (simulated by the model) and actual statewide *influenza-like hospitalizations*. Specifically, for each candidate network, we perform a least squares multilinear regression from the simulated ILINet time series to the actual Texas hospitalization time series, and use the coefficient of determination, 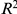, as the indicator of ILINet performance. Henceforth, we will refer to these models as *ILINet regression models*.

**Figure 1:**
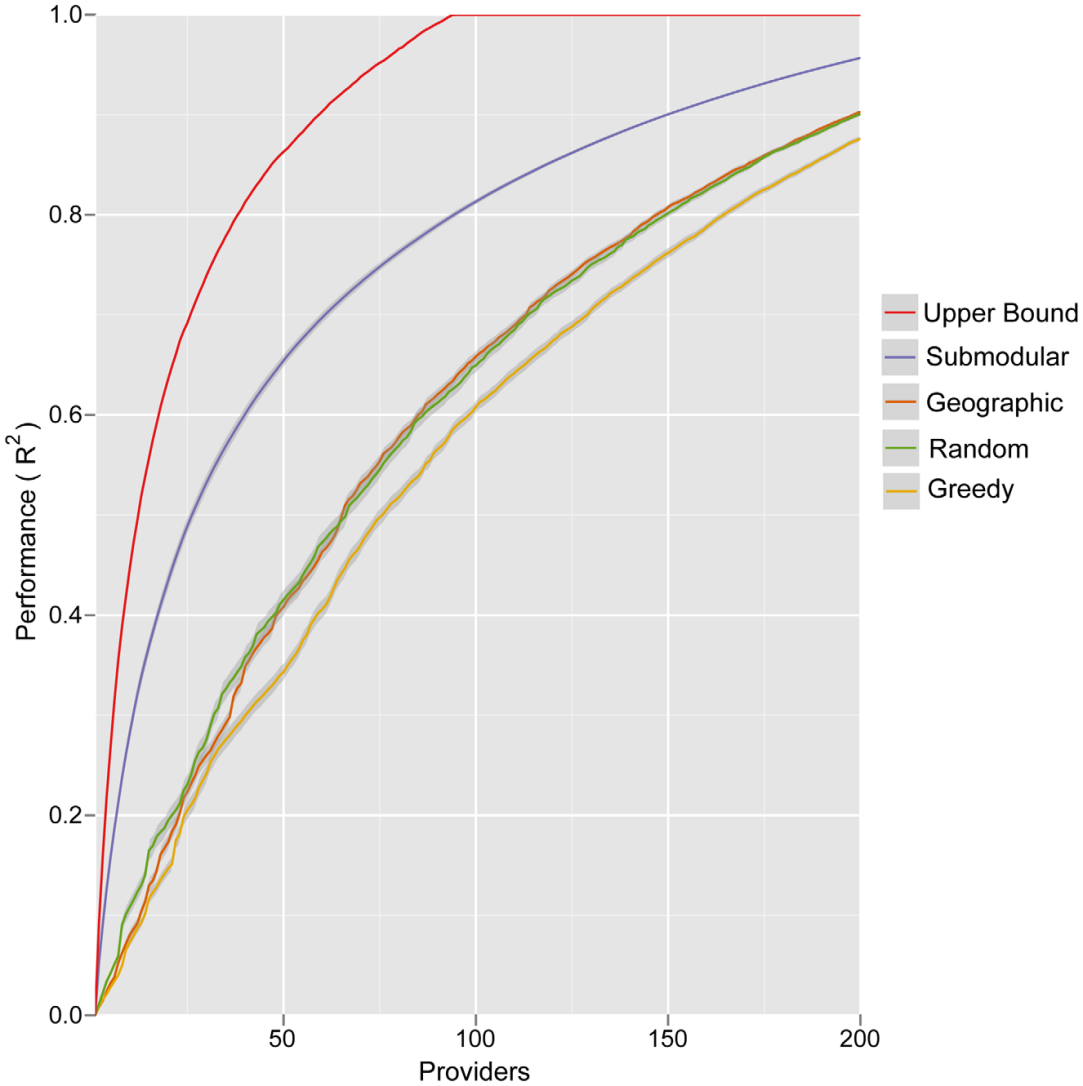
Expected performance of optimized ILINets. Four different methods were used to design Texas ILINets that effectively predict state-wide influenza hospitalizations. Submodular optimization (Submodular) outperforms random selection proportional to population density (Random), greedy selection strictly in order of population density (Greedy), and geographic optimization to maximize the number of people that live within 20 miles of a provider [17] (Geographic). The theoretical upper bound for performance (Upper Bound) gives the maximum 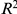 possible for a network designed by an exhaustive evaluation of all possible networks of a given size. For each network of each size, the following procedure was repeated 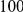 times: randomly sample a set of reporting profiles, one for each provider in the network; simulate an ILI time series for each provider in the network; perform an ordinary least squares multilinear regression from the simulated provider reports to the actual statewide influenza hospitalization data. The lines indicate the mean of the resulting 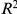 values, and the error bands indicate the middle 90% of resulting 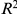 values, reflecting variation stemming from inconsistent provider reporting and informational noise.

**Figure 2:**
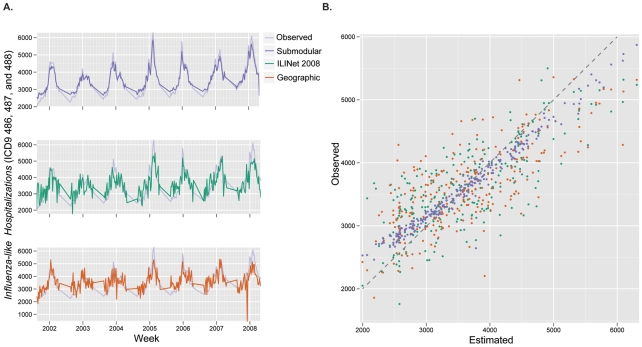
Comparing ILINet estimates to actual state-wide influenza hospitalizations. Statewide hospitalizations are estimated using data from three ILINets: the 2008 Texas ILINet (ILINet 2008), which consisted of 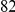 providers, and ILINets of the same size that were designed using submodular optimization (Submodular) and maximum coverage optimization with a 20 mile coverage distance (Geographic). (a) The estimates from each network are compared to actual Texas state-wide influenza hospital discharges from 2001–2008 (Observed). (b) The submodular ILINet yields estimates that are consistently closer to observed values than the other two ILINets. For each of the three networks, the following procedure was repeated 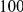 times: randomly sample a set of reporting profiles, one for each provider in the network; simulate an ILI time series for each provider in the network; perform an ordinary least squares multilinear regression from the simulated provider reports to the actual Texas influenza hospitalization data; and apply resulting regression model to the simulated provider time series data to produce estimates of statewide hospitalizations. The figures are based on averages across the 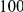 estimated hospitalization time series for each ILINet.

We compare the networks generated by this method to networks generated by two naive models and a published computational method [17] (Figure 1). *Random* selection models an open call for providers and entails selecting providers randomly with probabilities proportional to their zip code's population; *Greedy* selection prioritizes providers strictly by the population density of their zip code. Submodular optimization significantly outperforms these naive methods, particularly for small networks, with *Random* selection producing slightly more informative networks than *Greedy* selection. The *Geographic* optimization method of Polgreen et al. [17] selects providers to maximize the number of people that live within a specified “coverage distance” of a provider. Submodular optimization consistently produces more informative networks than this method at a 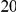 mile coverage distance (Figure 1) (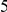, 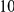, and 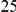 mile coverage distances perform worse, not shown). To visualize the relative performance of several of these networks, we compared their estimates of *influenza-like hospitalizations* (by applying each ILINet regression model to simulated ILINet report data) to the true state-wide hospitalization data (Figure 2). The time series estimated by a network designed using submodular optimization more closely and smoothly matches true hospitalizations than both the actual 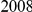 Texas ILINet and a network designed using geographic optimization (each with 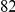 providers).

The submodular optimization algorithm is not guaranteed to find the highest performing provider network, and an exhaustive search for the optimal 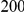 provider network from the pool of 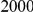 providers is computationally intractable. However, the submodular property of the objective function allows us to compute an upper bound on the performance of the optimal network, without knowing its actual composition (Figure 1). The performance gap between the theoretical upper bound and the optimized networks may indicate that the upper bound is loose (higher than the performance of the true optimal network) and/or the existence of better networks that might be found using more powerful optimization methods.

**Figure 3:**
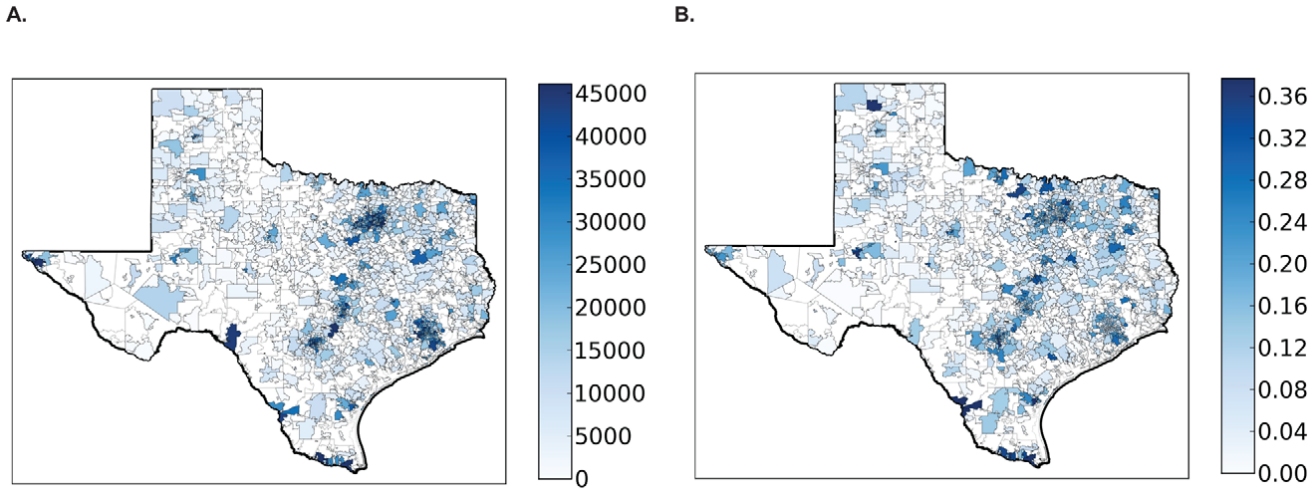
Statewide influenza activity mirrors population distribution. (a) Shading indicates zip code level population sizes, as reported in the 2000 census. (b) Major populations centers exhibit covariation in influenza activity. We performed a principal component analysis (PCA) on the centered hospitalization time series of all zip codes and calculated the time series of the first principal component. Zip codes are shaded according to the 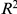 obtained from a regression of the first principal component time series to the influenza hospitalization time series for the zip code. Dark shading indicates high synchrony between influenza activity in the zip code and the first principal component. The correspondence between darkly shaded zip codes in (a) and (b) results from the high degree of synchrony in influenza activity between highly populated zip codes in Texas.

**Figure 4:**
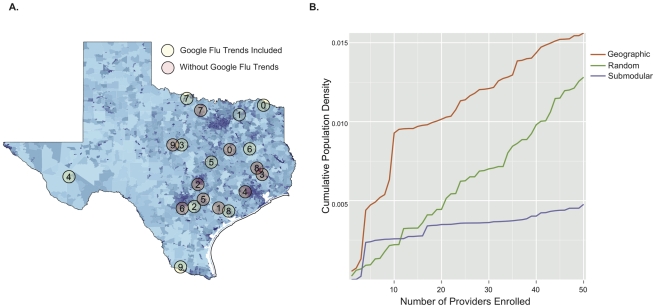
Location and population coverage of optimized ILINets. (a) Shading indicates zip code level population sizes, as reported in the 2000 census. Circles indicate the location (zip code) of the first ten providers selected when Google Flu Trends is included as a provider (green) and when it is not (pink). Numbers indicate selection order, with zero being the first provider selected and nine the tenth provider selected. (b) The cumulative population densities covered increase as each ILINet grows. Cumulative density is estimated by dividing total population of all provider zip codes by total area of all provider zip codes. While ILINets designed using the geographic (orange) and random (green) methods primarily target zip codes with high population densities, submodular optimization (purple) targets zip codes that provide maximal information, regardless of population density. All three networks cover approximately the same total number of people.

The networks selected by submodular optimization reveal some unexpected design principles. Most of the Texas population resides in Houston and the “I-35 corridor” – a North-South transportation corridor spanning San Antonio, Austin, and Dallas (Figure 3a). The first ten provider locations selected by submodular optimization are spread throughout the eastern half of the state (Figure 4a, pink circles). While most of the providers are concentrated closer to Texas' population belt, only two are actually located within Texas' major population centers (in this case, College Station).

The submodular networks are qualitatively different from the networks created by the other algorithms considered, which focus providers within the major population centers (Figure 4b). The higher performance of the submodular ILINets suggest that over-concentration of providers in major population centers is unnecessary. Influenza levels in the major population centers are strongly correlated (Figure 3b). Thus, ILINet information from San Antonio, for example, will also be indicative of influenza levels in Austin and Dallas. This synchrony probably arises, in part, from extensive travel between the major Texas population centers.

### Subsampling and Augmenting an ILINet

**Figure 5:**
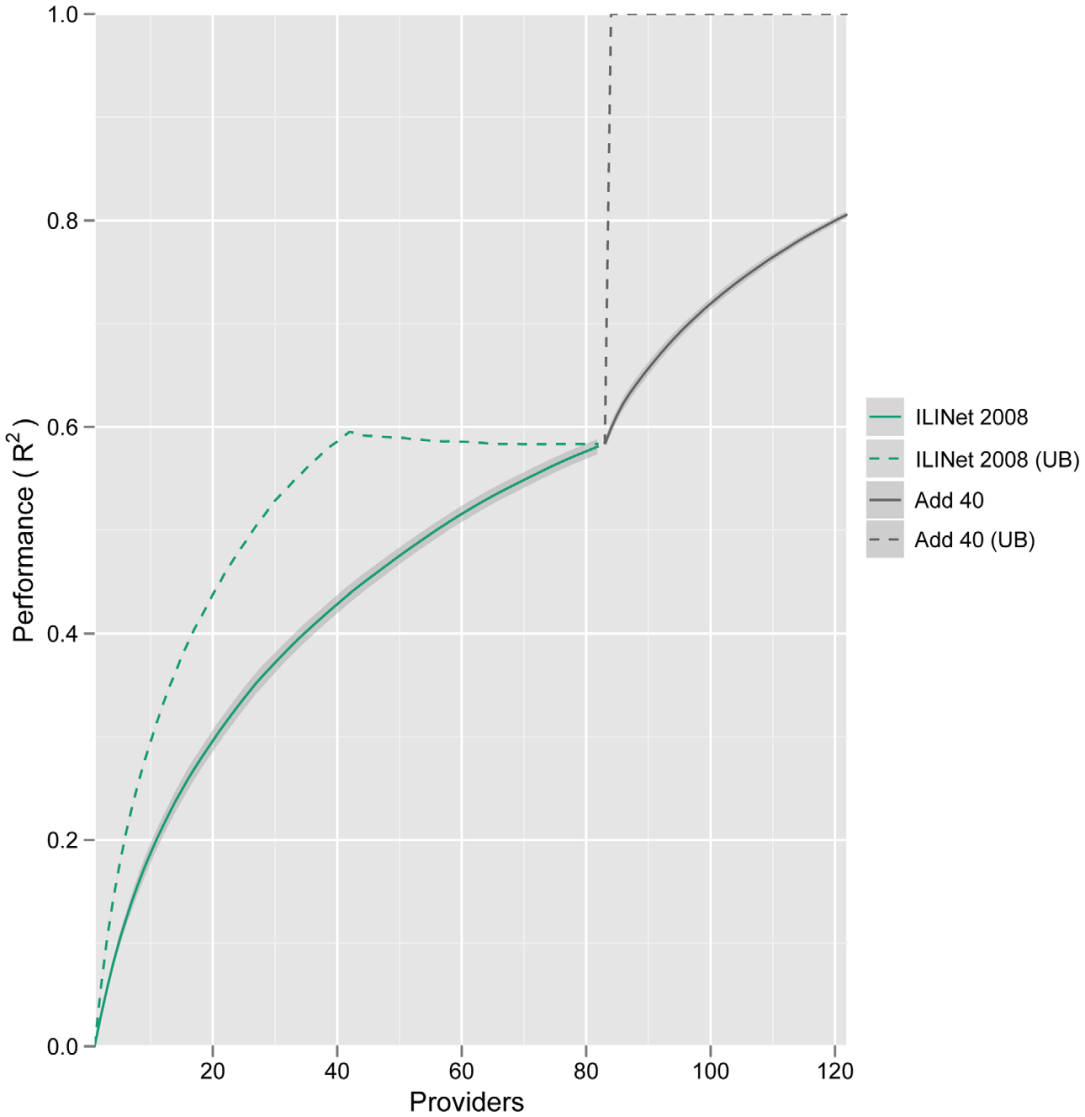
Augmenting an existing ILINet. This compares theoretical upper bounds (dashed lines) to the performance of a submodular optimized ILINet built by first subsampling the 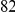 zip codes of providers actually enrolled in Texas' 2008 ILINet (green) and then adding 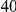 additional providers from elsewhere in the state (gray). The error bands indicate the middle 90% of resulting 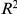 values, and reflect variation stemming from inconsistent provider reporting rates and informational noise.

Using submodular optimization, we augment the 2008 Texas ILINet by first subsampling from the 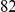 enrolled providers and then adding up to 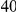 new providers. When subsampling, performance does not reach a maximum until all 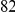 providers are included in the network (Figure 5), indicating that each provider adds predictive value to the network. However, the theoretical upper bound plateaus around 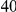 providers, suggesting that smaller (more optimally chosen) networks of equal predictive value may exist. During the second stage, 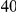 additional providers improve the 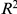 objective by 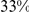. Most of these providers are located in relatively remote areas of the state.

**Figure 6:**
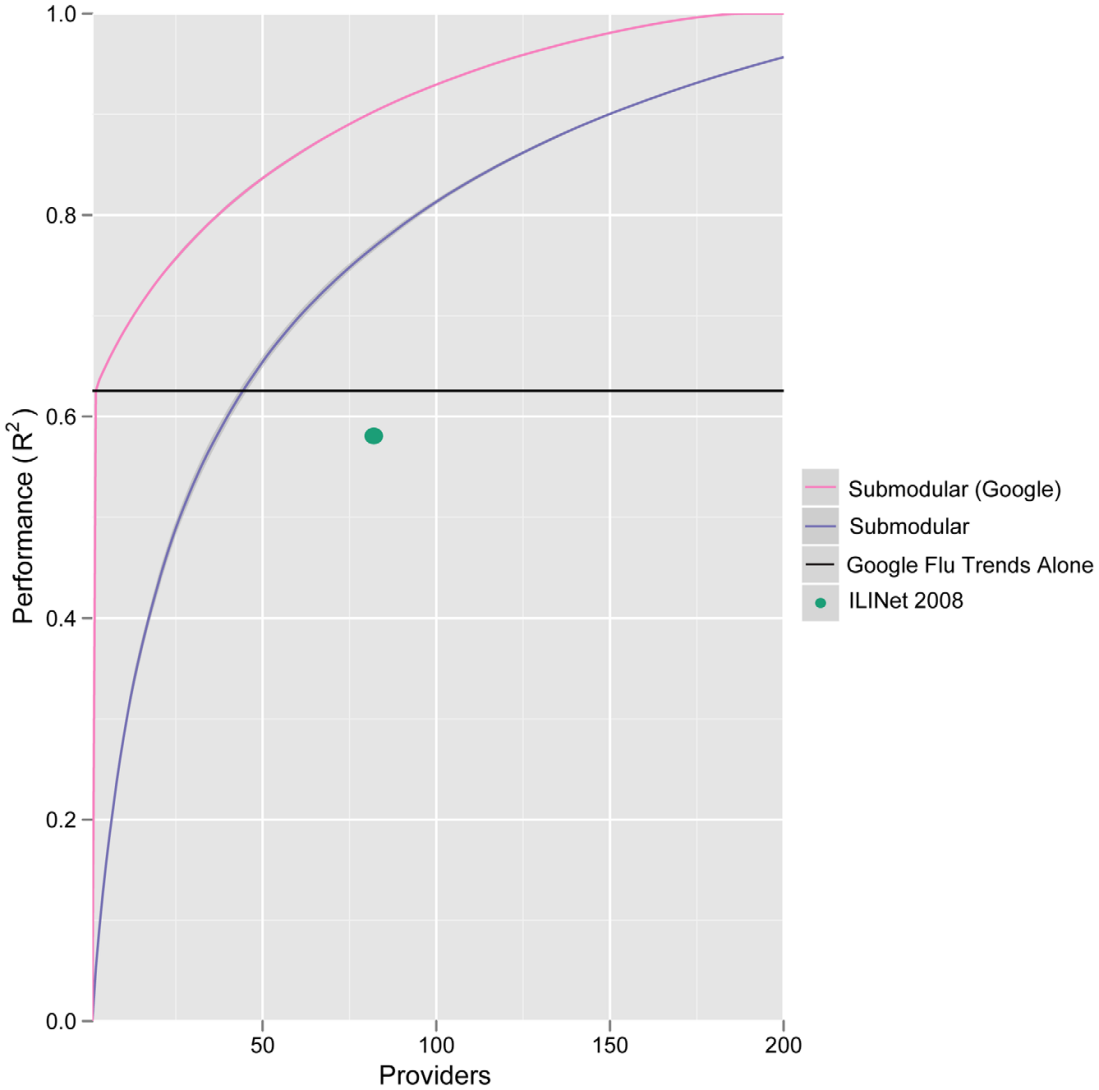
Google Flu Trends as a virtual ILINet provider. When state-level Google Flu Trends is treated as a possible provider, submodular optimization choses it as the first (most informative) provider for the Texas ILINet, and results in a high performing network (pink line). Alone (black line), the Google Flu Trends provider performs as well as a traditional submodular optimized network (blue line) containing 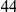 providers (intersection of black and purple lines) and outperforms the actual 2008 Texas ILINet (green dot).

We also considered inclusion of Internet trend data sources as virtual providers, specifically, the freely available Google Flu Trends data for the state of Texas [21]. Google Flu Trends alone is able to explain about 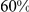 of the variation in state-wide hospitalizations; it outperforms the 2008 Texas ILINet and matches the performance of a network with 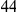 traditional providers constructed from scratch using submodular optimization (Figure 6). However, the best networks include both traditional providers and Google Flu Trends. For example, by adding 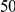 providers to Google Flu Trends using submodular optimization, we improve the 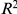 objective by a third and halve the optimality gap (from a trivial upper bound of one). The additional providers are located in non-urban areas (Figure 4a, green circles) distinct from those selected when Google Flu Trends is not allowed as a provider.

### Out-of-Sample Validation

**Figure 7:**
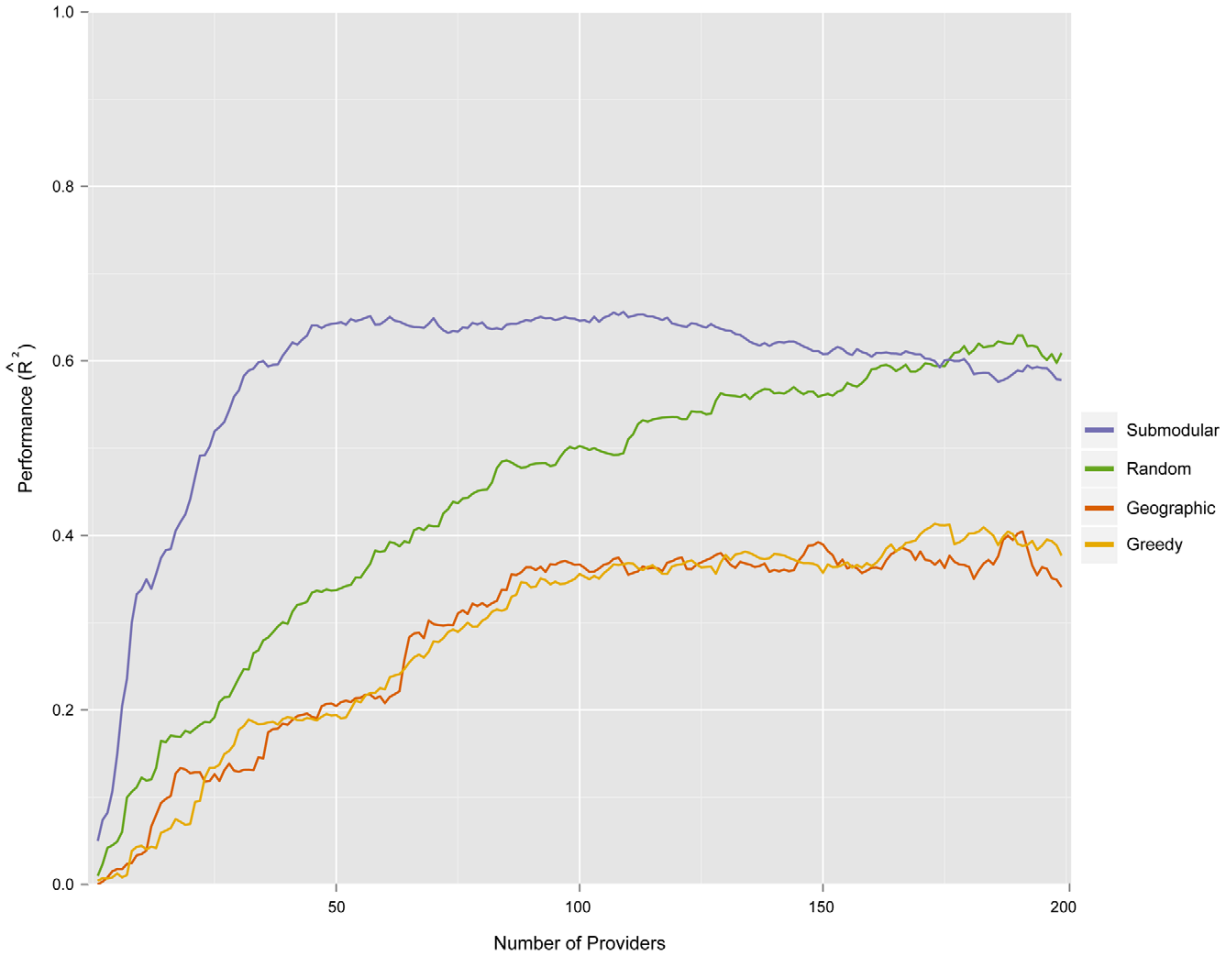
Predictive performance of ILINets. Data from the 2001–2007 period were used to design ILINets and estimate multilinear regression prediction models. The predictive performance of the ILINets (y-axis) is based on a comparison between the models' predictions for 2008 hospitalizations (from mock provider reports) and actual 2008 hospitalization data. For almost all network sizes, Submodular optimization (Submodular) outperforms random selection proportional to population density (Random), greedy selection strictly in order of population density (Greedy), and geographic optimization to maximize the number of people that live within 20 miles of a provider [17] (Geographic). The leveling-off of performance around 100 providers is likely a result of over-fitting, given that there were only 222 historical time-points used to estimate the original model.

To further validate our methodology, we simulated the real-world scenario in which historical data are used to design an ILINet and build forecasting models, and then current ILINet reports are used to make forecasts. Specifically, we used 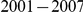 data to design ILINets and estimate multilinear regression models relating *influenza-like hospitalizations* to mock provider reports, and then used 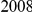 data to test the models' ability to forecast *influenza-like hospitalizations*. For networks with fewer than 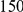 providers, the ILINets designed using submodular optimization consistently outperform ILINets designed using the other three strategies (Figure 7). Above 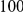 providers, the predictive performance of the submodular optimization ILINet begins to decline with additional providers. As the number of providers approaches 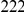 (the number of weeks in the training period), the estimated prediction models become overfit to the 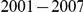 period. Thus, the slightly increased performance of the *Random* method over the submodular optimization after 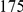 providers is spurious. For the 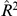 values presented in Figure 7, the effect of noise and variable reporting are integrated out when calculating the expected provider reports. An alternative approach to out-of-sample validation is presented in Text S1; it yields the same rank-order of model performance.

## Discussion

Since the mid twentieth century, influenza surveillance has been recognized as an increasingly complex problem of global concern [22]. However, the majority of statistical research has focused on the analysis of surveillance data rather than the data collection itself, with a few notable exceptions [12], [17]. High quality data is essential for effectively monitoring seasonal dynamics, detecting anomalies, such as emerging pandemic strains, and implementing effective time-sensitive control measures. Using a new method for optimizing provider-based surveillance systems, we have shown that the Texas state ILINet would benefit from the inclusion of a few strategically selected providers and the use of Internet data streams.

Our method works by iteratively selecting providers that contribute the most information about *influenza-like hospitalizations*. We quantified the performance of various ILINets using the coefficient of determination 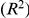 resulting from a multi-linear regression between each provider's time series and state-wide *influenza-like hospitalizations*. Importantly, these simulated providers have reporting rates and error distributions estimated from actual ILINet providers in Texas (see Text S1). The result is a prioritized list of zip codes for inclusion in an ILINet that can be used for future ILINet recruiting. Although this analysis was specifically motivated by the Texas DSHS interest in predicting hospitalizations with ICD9 codes 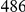, 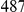, and 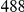, our method can be readily extended to design a network for any disease or influenza definition with the appropriate historical data. In general, the method requires both historical provider reports and historical time series of the prediction target. However, if one has reasonable estimates of provider reporting rates and informational noise from another source (e.g., estimates from a surveillance network in another region or for another disease), then historical provider reports are not necessary.

ILINet provider reports do not necessarily reflect true influenza activity. Rather they are supposed to indicate the number of patients that meet the clinical ILI case definition, which results in a substantial number of false positives (reported non-influenza cases) and false negatives (missed cases of influenza) [23]. The case definition for ILI is often loosely applied, further confounding the relationship between these measures and true influenza. Similarly, the ICD9 codes used in our analysis do not correspond perfectly to influenza hospitalizations: some influenza cases will fail to be classified under those codes, and some non-influenza cases will be. Nonetheless, public health agencies are interested in monitoring and forecasting the large numbers of costly hospitalizations associated with these codes. We find that ILINet surveillance data correlates strongly with this set of *influenza-like hospitalizations*, and that the networks can be designed to be even more informative.

Although we provide only a single example here, this optimization method can be readily applied to designing surveillance networks for a wide range of diseases on any geographic scale, provided historical data are available and the goals of the surveillance network can be quantified. For example, surveillance networks could be designed to detect emerging strains of influenza on a global scale, monitor influenza in countries without surveillance networks, or track other infectious diseases such as malaria, whooping cough, or tuberculosis or non-infectious diseases and chronic conditions such as asthma, diabetes, cancer or obesity that exhibit heterogeneity in space, time or by population subgroup. As we have shown with Google Flu Trends, our method can be leveraged to evaluate the potential utility of incorporating other Internet trend data mined from search, social media, and online commerce platforms into traditional surveillance systems.

While optimized networks meet their specified goals, they may suffer from over optimization and be unable to provide valuable information for other diseases or even for the focal disease during atypical situations. For example, a surveillance network designed for detecting the early emergence of pandemic influenza may look very different from one optimized to monitor seasonal influenza. Furthermore, an ILINet optimized to predict *influenza-like hospitalizations* in a specific socio-economic group, geographic region, or race/ethnicity may look very different from an ILINet optimized to predict state-wide hospitalizations. When optimizing networks, it is thus important to carefully consider the full range of possible applications of the network and integrate diverse objectives into the optimization analysis.

The optimized Texas ILINets described above exhibit much less redundancy (geographic overlap in providers) than the actual Texas ILINet. Whereas CDC guidelines have led Texas DSHS to focus the majority of recruitment on high population centers, the optimizer only sparsely covered the major urban areas because of their synchrony in influenza activity. This is an important distinction between submodular optimization and the other methods considered (*Geographic*, *Random* and *Greedy*). The submodular method does not track population density and instead adds providers who contribute the most marginal information to the network. Consequently, it places far more providers in rural areas than the other methods (Figure 4b). There can be substantial year-to-year variation in spatial synchrony for seasonal influenza, driven by the predominant influenza strains and commuter traffic between population centers [24]. As long as the historical data used during optimization reflect this stochasticity, the resulting networks will be robust. However, synchrony by geography and population density does not occur for all diseases including emerging pandemic influenza [24]; thus the relatively sparse networks designed for forecasting seasonal influenza hospitalizations may not be appropriate for other surveillance objectives, like detecting emerging pandemic strains or other rare events. For example, a recent study of influenza surveillance in Beijing, PRC suggested that large hospitals provided the best surveillance information for seasonal influenza, while smaller provincial hospitals were more useful for monitoring H5N1 [12].

Although our method outperforms the *Maximal Coverage Method* (MCM), referred to as *Geographic*, proposed by Polgreen et al. (2009), there are several caveats. First, population densities and travel patterns within Texas are highly non-uniform. The two methods might perform similarly for regions with greater spatial uniformity. Second, our method is data intensive, requiring historical surveillance data that may not be available, for example, in developing nations, whereas the population density data required for MCM is widely available. However, the type of data used in this study is readily available to most state public health agencies in the United States. For example, the CDC's Influenza Hospitalization Network (FluSurv-NET) collects weekly reports on laboratory confirmed influenza-related hospitalizations in fourteen states. In addition, alternative internet-based data sources like Google Flu Trends are becoming available. Third, as discussed above, our networks are optimized towards specific goals and may thus have no expected level of performance for alternate surveillance goals. Important future research should focus on designing networks able to perform well under a range of surveillance goals. Fourth, neither ILINet data nor *influenza-like hospitalizations* correspond perfectly to actual influenza activity. One could instead optimize ILINets using historical time series of laboratory-confirmed cases of influenza. Although some provider locations and the estimated regression models may change, we conjecture that the general geospatial distribution of providers will not change significantly. Fourth, we followed Polgreen et al. (2009)'s use of Euclidean distances. However, travel distance is known to correlate more strongly with influenza transmission than Euclidean distance [24], and thus alternative distance metrics might improve the performance of the MCM method. Finally, while submodular optimization generally outperforms the other design methods in out-of-sample prediction of *influenza-like hospitalizations*, it suffers from overfitting when the number of providers in the network approaches the number of data points in the historical time series.

The impressive performance of Google Flu Trends leads us to question the role of traditional methods, such as provider-based surveillance networks, in next generation disease surveillance systems. While Texas Google Flu Trends alone providers almost as much information about state-wide influenza hospital discharges as the entire 2008 Texas ILINet, an optimized ILINet of the same size contains 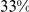 more information than Google Flu Trends alone. Adding Google Flu Trends to this optimized network as a virtual provider increases its performance by an additional 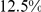. Internet driven data streams, like Google Flu Trends, may have age and socio-economic biases that over-represent certain groups, a possible explanation for the difference in providers selected when Google Flu Trends is included, Figure 4a. Given the relatively low cost of voluntary provider surveillance networks, synergistic approaches that combine data from conventional and Internet sources offer a promising path forward for public health surveillance.

This optimization method was conceived through a collaboration between The University of Texas at Austin and the Texas Department of State Health Services to evaluate and improve the Texas ILINet. The development and utility of quantitative methods to support public health decision making hinges on the continued partnership between researchers and public health agencies.

## Materials and Methods

### Data

The Texas Department of State Health Services (DSHS) provided (1) ILINet data containing weekly records from 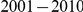 reporting the number of patients with influenza-like-illness and the total number of patients seen by each provider in the network, and (2) individual discharge records for every hospital in Texas from 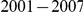 (excluding hospitals in counties with less than 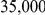 inhabitants, in counties with less than 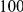 total hospital beds, or those hospitals that do not seek insurance payment or government reimbursement). We classified all hospital discharges containing ICD9 codes of 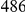, 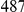, or 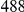 as influenza-related. Google Flu Trends data was downloaded from the Google Flu Trends site [21] and contains estimates of ILI cases per 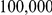 physician visits determined using Google searches [25]. Data on population size and density was obtained from the 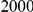 census [26].

### Provider Reporting Model

The first step in the ILINet optimization is to build a data-driven model reflecting actual provider reporting rates and informational noise, that is, inconsistencies between provider reports and true local influenza prevalence.

We model reporting as a Markov process, where each provider is in a “reporting” or “non-reporting” state. A provider in the reporting state enters weekly reports, while a provider in the non-reporting state does not enter reports. At the end of each week, providers independently transition between the reporting and non-reporting states. Such a Markov process model allows for streaks of reporting and streaks of non-reporting for each provider, which is typical for ILINet providers. We estimate transition probabilities between states from actual ILINet provider report data. For each provider, the transition probability from reporting to non-reporting is estimated by dividing the number of times the transition occurred by the number of times any transition out of reporting is observed. The probabilities of remaining in the current reporting state and transitioning from non-reporting to reporting are estimated similarly.

We model noise in reports using a standard regression noise model of the form
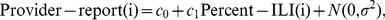
(1)where 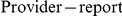
(*i*) denotes the number of ILI cases reported by the provider in week 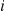; 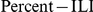
(*i*) denotes the estimated prevalence of ILI in the provider's zip code in week 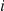; 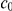 and 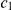 are regression constants fixed for the provider; and 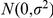 is a normally distributed noise term with variance 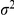 also fixed for the provider. For existing providers, we use empirical time series (their past ILINet reporting data matched with local ILI prevalence, described below) to estimate the constants 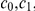 and 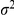 using least squares linear regression. This noise model has the intuitive interpretation that each provider's reports are a noisy reading of the percent of the population with ILI in the provider's zip code.

We use the Texas hospital discharge data to estimate the local ILI prevalences (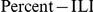
(*i*)) for each zip code. Given an estimate of the influenza hospitalization rate [27] and assuming that each individual with ILI is hospitalized independently, we can obtain a distribution for the number of influenza-related hospitalizations in a zip code, given the number of ILI cases in the zip code. Using Bayes rule, a uniform prior, and the real number of influenza-related hospitalizations (from the hospital discharge data), we derive distributions for the number of ILI cases for each zip code and each week. We then set 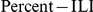
(i) for each zip code equal to the mean of the distribution of ILI cases in that zip code for week 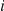, divided by the population of the zip code.

### Generating Pools of Mock Providers

The second step in the ILINet optimization is to generate a pool of mock providers. For each actual provider in the Texas ILINet, we estimate a reporting profile specified by [1)] transition probabilities between reporting and non-reporting (Markov) states, and the constants 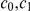 and 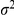, modeling noise in the weekly ILI reports. To generate a mock provider in a specified zip code, we select a uniformly random reporting profile out of all reporting profiles estimated from existing providers. The generated mock providers are thereby given reporting characteristics typical of existing providers. We can then generate an ILI report time series for a mock provider, by 1) generating reports only during reporting weeks, and calculating reports using equation (1) with the constants given in the provider's reporting profile and estimates of 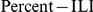
(*i*) for the mock provider's zip code.

We select providers from pools consisting of a single mock provider from each zip code. Zip codes offer a convenient spatial resolution, because they have geographic specificity and are recorded in both the Texas ILINet and hospital discharge data. The optimization algorithm is not aware of a mock provider's reporting profile when the provider is selected (discussed below).

### Provider Selection Optimization

The final step in our ILINet design method is selecting an optimized subset of providers from the mock provider pool. We seek the subset that most effectively predicts a target time series (henceforth, goal), as measured by the coefficient of determination (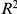) from a least squares multilinear regression to the goal from the report time series for all providers in the subset. Specifically, the objective function is given by
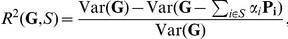
where 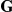 is the goal random variable; 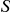 is a subset of the mock provider pool; 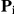 are provider reports for provider 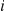; and the 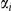 are the best multilinear regression coefficients (values that minimize the second term in the numerator).

There are several advantages to this objective function. First, it allows us to optimize an ILINet for predicting a particular random variable. Here, we set the goal to be state-wide influenza-related hospitalizations for Texas. This method can be applied similarly to design surveillance networks that predict, for example, morbidity and/or mortality within specific age groups or high risk groups.

Second, the objective function is submodular in the set of providers, 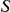 [28], implying generally that adding a new provider to a small network will improve performance more than adding the provider to a larger network. The submodular property enables computationally efficient searches for near optimal networks and guarantees a good level of performance from the resulting network [29]. Without a submodular objective function, optimization of a 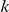 provider ILINet may require an exhaustive search of all subsets of 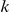 providers from the provider pool, which quickly becomes intractable. For example, an exhaustive search for the optimal 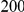 provider Texas ILINet from our pool of approximately 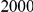 mock providers would require roughly 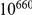 regressions.

Taking advantage of the submodular property, we rapidly build high performing networks (with 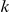 providers) according to the following algorithm:

1. Let 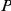 be the entire provider pool, 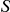 be the providers selected thus far, and 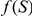 be a submodular function in 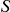. We begin without any providers in 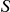.
2. Repeat until there are 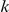 providers in 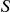:
  - Let 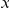 be the provider in 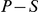 that maximizes 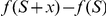

  - Add 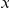 to 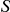.

This is guaranteed to produce a network that performs within a fraction of 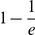 of the optimal network [28]. The submodularity property also allows us to compute a posterior bound on the distance from optimality, which is often much better than 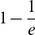. Finally, even if implemented naively, the algorithm only requires approximately 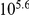 regressions to select 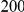 providers from a pool of 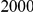.

When optimizing, it is important to consider potential noise (underreporting and discrepancies between provider reports and actual ILI activity in the zip code). However, we assume that one cannot predict the performance of a particular provider before the provider is recruited into the network. To address this issue, the optimization's objective function is an expectation over the possible provider reporting profiles. Specifically, we define 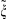 as a random variable describing the provider reporting profile for the entire pool of mock providers. If 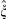 is a specific reporting profile, then the 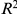 objective function can be written as
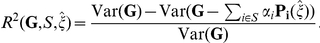
To design the ILINet, we solve the following optimization problem
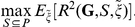
The objective function is a convex combination of submodular functions, and thus is also submodular. This allows us to use the above algorithm along with its theoretical guarantees to design ILINets using a realistic model of reporting practices and informational stochasticity, without assuming that the designer knows the quality of specific providers *a priori*.

### Maximal Coverage Model

We implemented the *Maximal coverage model* (MCM) following Polgreen et al. (2009). Briefly, a greedy algorithm was used to minimize the number of people in Texas who live outside a pre-defined coverage distance, 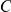, of at least one provider in the selected set, 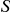. A general version of this algorithm was developed by Church and Re Velle (1974) to solve this class of MCM's [30]. As per Polgreen et al. (2009), we assumed that the population density of each zip code exists entirely at the geographic center of the zip code and used Euclidean distance to measure the distance between zip codes. Using a matrix of inter-zip code distances we select providers iteratively, choosing zip codes that cover the greatest amount of population density within the pre-defined coverage distance, 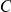. We considered 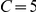, 10, 20, and 25 miles, and found that 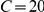 miles yielded the most informative networks.

### Naive Methods

We used two naive methods to model common design practices for state-level provider-based surveillance networks.

1. *Greedy selection by population density* - All zip codes were ordered by population density and added to the provider pool 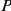. Providers are then moved from 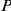 to the selected set 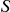 from highest to lowest density. The algorithm stops when 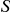 reaches a pre-determined size or 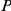 is empty.
2. *Uniform random by population size* - Zip codes are randomly selected from 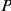 and moved to 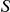 with a probability proportional to their population size. The algorithm proceeds until either 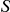 reaches a pre-determined size or 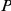 is empty.

### Principal Component Analysis of Hospitalization Time Series across Texas Zip Codes

To analyze similarities in ILI hospitalizations across different zip codes, we apply principal component analysis (PCA) [31]. Specifically, we perform PCA on the centered (mean zero), standardized (unit variance) hospitalization time series of all zip codes in Texas. We first compute a time series for the first principal component, and then compute an 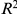 for each zip code, based on a linear regression from the first principal component to the zip code's centered, standardized hospitalizations. Zip codes with high 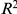 values have hospitalization patterns that exhibit high temporal synchronicity with the first principal component.

### Out-of-Sample Validation

To validate our method, we first use submodular optimization to create a provider network of 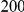 providers, using only data from 2001 to 2007, and then evaluate the performance of the network in predicting 2008 influenza-like hospitalizations. Specifically, after creating the 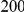-provider network (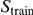), we use actual hospitalization data and mock provider reports for the 2001–2007 period to fit a multilinear regression model of the form 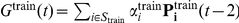 where 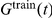 is time series of state-wide influenza-like hospitalizations at week 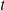 for weeks in 2001 to 2007, 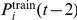 is the mock report time series of provider 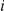 during week 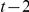 for weeks in 2001 to 2007, and 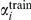 is the best multilinear regression coefficient associated with provider 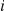.

We then use the estimated multilinear regression function to forecast state-wide influenza-like hospitalization during 2008 from mock provider reports of 2008, and compare these forecasts to actual 2008 hospitalization data. This simulates a real-world prediction, where only historical data is available to create the provider network (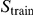) and estimate the prediction function (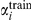
's), and then the most recent provider reports (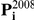
's) are used to make predictions. We evaluate the 2008 predictions using a variance reduction measure similar to 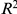, except that the multilinear prediction model uses coefficients estimated from prior data, as given by
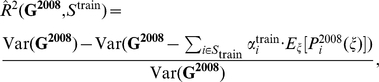
where 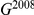 is the hospitalization time series in 2008, 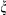 is the provider noise profile, and 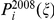 are the mock provider reports in 2008. Importantly, we first calculate an expected value for the provider reports, 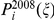, given the noise profiles 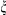, before calculating 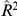. We also considered an alternative validation method in which we first calculate an 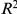 for each provider report and noise-profile combination, and then analyze the resulting distribution of 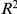 values (see Text S1 for results).

## Supporting Information

Figure S1 **Proportion of hospitalizations associated with ICD9s 486, 487 and 488 -** We present the proportion of respiratory illness related hospitalizations that were also associated with ICD9s 486, 487 and 488. The total number of respiratory illness related hospitalizations were estimated from the Texas hospitalization database, the same database used to determine the number of ICD9 486, 487 and 488 associated cases. There is a strong seasonality in the proportion, with peaks in the winter between 0.30 and 0.37 and valleys in the summer around 0.24.
(TIF)

Figure S2 **Weekly costs associated with ICD9s 486, 487 and 488 -** The total weekly billing charges associated with *influenza-like hospitalizations* are plotted from the end of 2001 through the beginning of 2009. On average 500 million dollars of hospital charges were billed per month to patients associated with ICD9s 486, 487 and 488. However, it is important to note the over two-fold increase in this amount since 2002. For the 2007–2008 influenza season this increase corresponded to a total billed amount of 9.3 billion dollars. This represents nearly 1 percent of the yearly GDP in Texas, which is not much less than the year-to-year economic growth.
(TIF)

Figure S3 **Texas ILINet provider reporting rates -** (a) Histograms are presented for the four transition probabilities used in our Markov model of provider reporting. The change in skew between panels *i* and *iv* as compared to panels *ii* and *iii* is expected given the observation of “streaky” reporting of ILINet providers in Texas. The providers with a score of one in panel *ii* are those ideal providers who are likely to resume reporting after missing a week. (b) A scatter plot of the values in S3a- *i* and S3a- *ii*, Report given Reported and Report given Failed to Report, are presented to indicate that there are both reliable and unreliable providers enrolled in the Texas ILINet, with darker blue indicating a more reliable provider and light-blue to white a less reliable provider.
(TIF)

Figure S4
**Out-of-Sample Model Validation -** We used data from 2001–2007 to design ILINets and to fit multi-linear prediction functions, and then generated provider-report based forecasts of hospitalizations during 2008 (without using any data from 2008) and compared these predictions to actual 2008 hospitalization data (see text for details). The 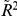 values reflect the predictive performance of the different ILINets. For each ILINet, we predicted 100 time series from simulated provider reports, each time drawing random deviates from the provider noise and reporting distributions, and then compared them to actual 2008 hospitalizations by calculating 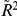. Lines indicate the average 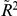 and shaded regions indicate the middle 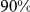 of the 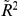 distribution. Negative values indicate that the predicted hospitalization time series are more variable than the actual time series. The increasingly poor performance and uncertainty with additional providers is a result of over-fitting of the prediction model to data from the 2001–2007 training period. The submodular method is the only one to yield ILINets with a 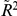 greater than zero.(TIF)

Figure S5 **The importance of realistic reporting rates and noise -** We compared the first ten providers selected by the submodular optimization method when providers either contained (a) perfect information and perfect reporting rates or (b) were subject to the patterns of imperfect and variable reporting exhibited by actual ILINet providers. When simulated providers had reporting probabilities and noise similar to actual providers the resulting network contained more geographic redundancy than one built from simulated providers with perfect information and reporting rates. All results presented in the manuscript were determined using simulated providers with patterns of imperfect and variable reporting derived from actual ILINet data. The stark difference highlights the importance of incorporating the characteristics of actual ILINet provider reporting.
(TIF)

Text S1 **In text S1, we present the results of five supplementary analyses.** 1) The importance of *influenza-like hospitalizations* in terms of total respiratory disease related hospitalizations and health care charges in Texas, 2) The details of actual ILINet provider reporting in Texas, the data described here were used to derive our provider reporting model, 3) The time-lagged, linear relationship between *influenza-like hospitalizations*, ILINet, and Google Flu Trends in Texas, 4) Additional model validation results, which support and confirm those presented in the main text, and 5) The importance of incorporating realistic provider reporting rates and noise illustrated by the dramatic difference in the results when perfect information and reporting is assumed.
(PDF)
