# Supplementary figures and images for: Optimizing Provider Recruitment for Influenza Surveillance Networks
Source: PLoS Comput Biol. 2012 Apr 12;8(4):e1002472. doi: 10.1371/journal.pcbi.1002472 (PMC3325176; doi:10.1371/journal.pcbi.1002472)

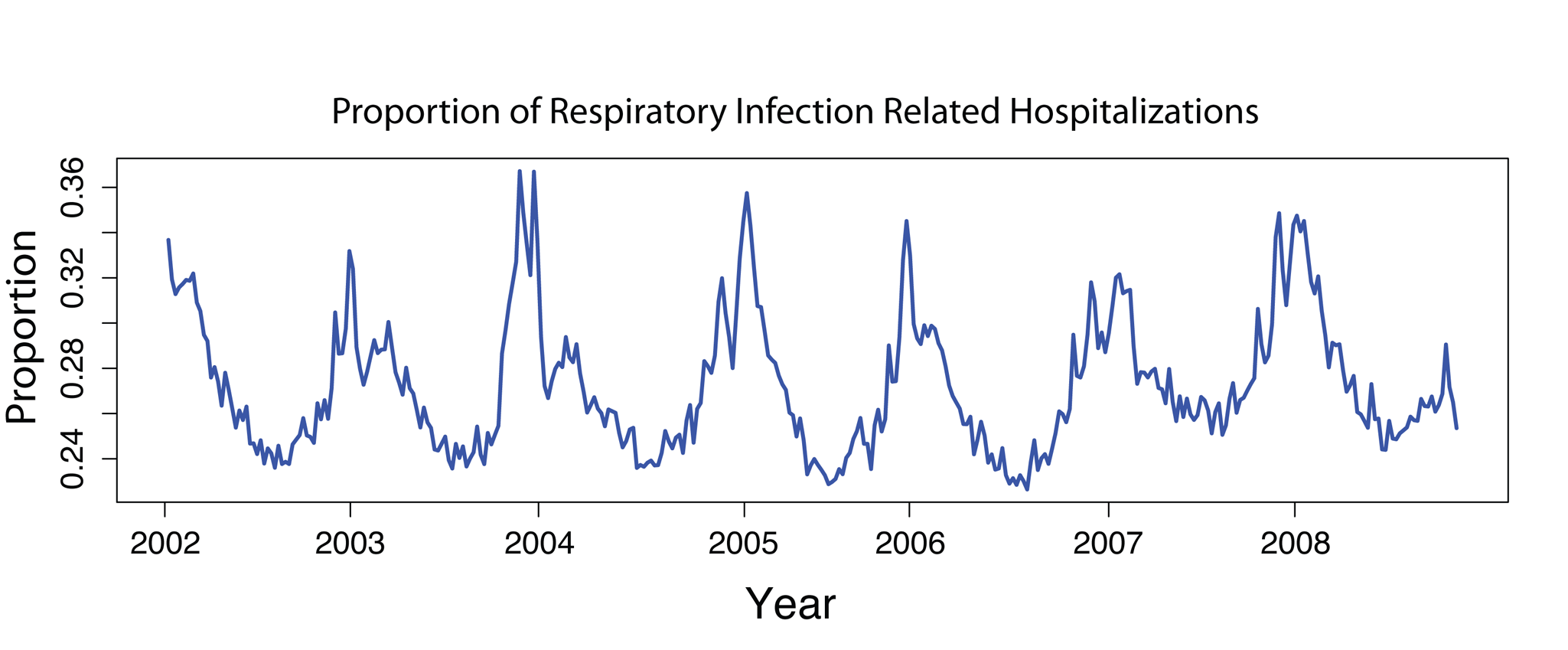

Supplement: Figure S1 — Proportion of hospitalizations associated with ICD9s 486, 487 and 488 - We present the proportion of respiratory illness related hospitalizations that were also associated with ICD9s 486, 487 and 488. The total number of respiratory illness related hospitalizations were estimated from the Texas hospitalization database, the same database used to determine the number of ICD9 486, 487 and 488 associated cases. There is a strong seasonality in the proportion, with peaks in the winter between 0.30 and 0.37 and valleys in the summer around 0.24. (TIF) [file pcbi.1002472.s001.tif]

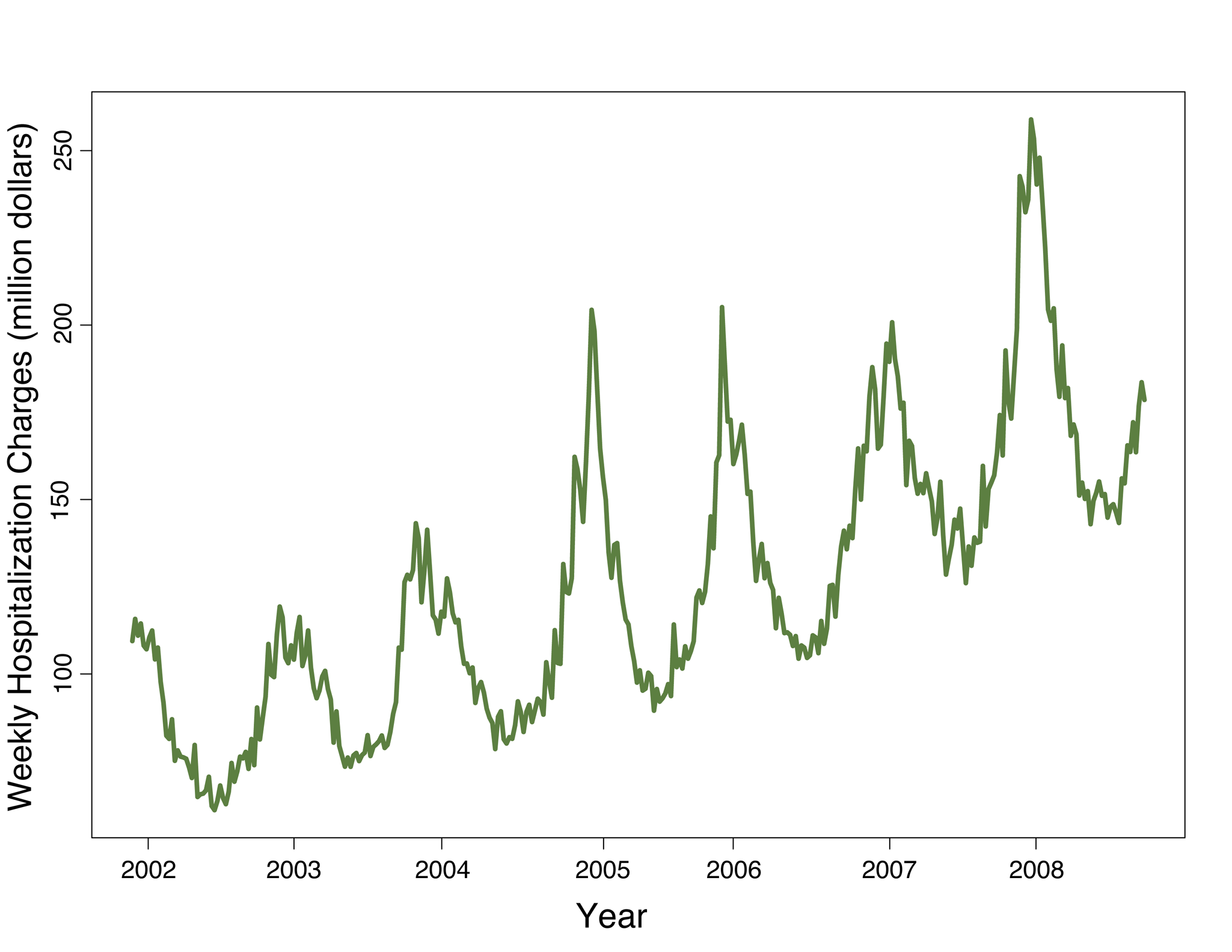

Supplement: Figure S2 — Weekly costs associated with ICD9s 486, 487 and 488 - The total weekly billing charges associated with influenza-like hospitalizations are plotted from the end of 2001 through the beginning of 2009. On average 500 million dollars of hospital charges were billed per month to patients associated with ICD9s 486, 487 and 488. However, it is important to note the over two-fold increase in this amount since 2002. For the 2007–2008 influenza season this increase corresponded to a total billed amount of 9.3 billion dollars. This represents nearly 1 percent of the yearly GDP in Texas, which is not much less than the year-to-year economic growth. (TIF) [file pcbi.1002472.s002.tif]

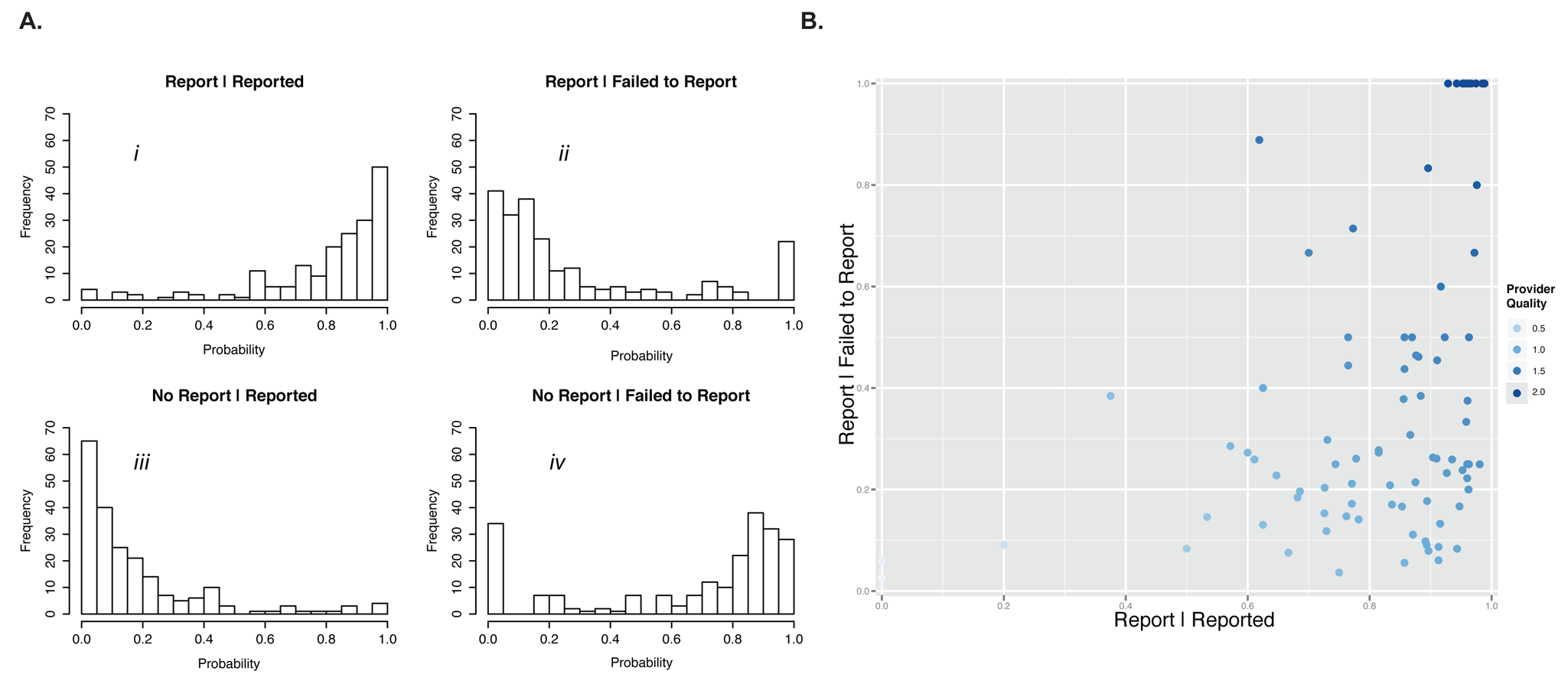

Supplement: Figure S3 — Texas ILINet provider reporting rates - (a) Histograms are presented for the four transition probabilities used in our Markov model of provider reporting. The change in skew between panels i and iv as compared to panels ii and iii is expected given the observation of “streaky” reporting of ILINet providers in Texas. The providers with a score of one in panel ii are those ideal providers who are likely to resume reporting after missing a week. (b) A scatter plot of the values in S3a- i and S3a- ii, Report given Reported and Report given Failed to Report, are presented to indicate that there are both reliable and unreliable providers enrolled in the Texas ILINet, with darker blue indicating a more reliable provider and light-blue to white a less reliable provider. (TIF) [file pcbi.1002472.s003.tif]

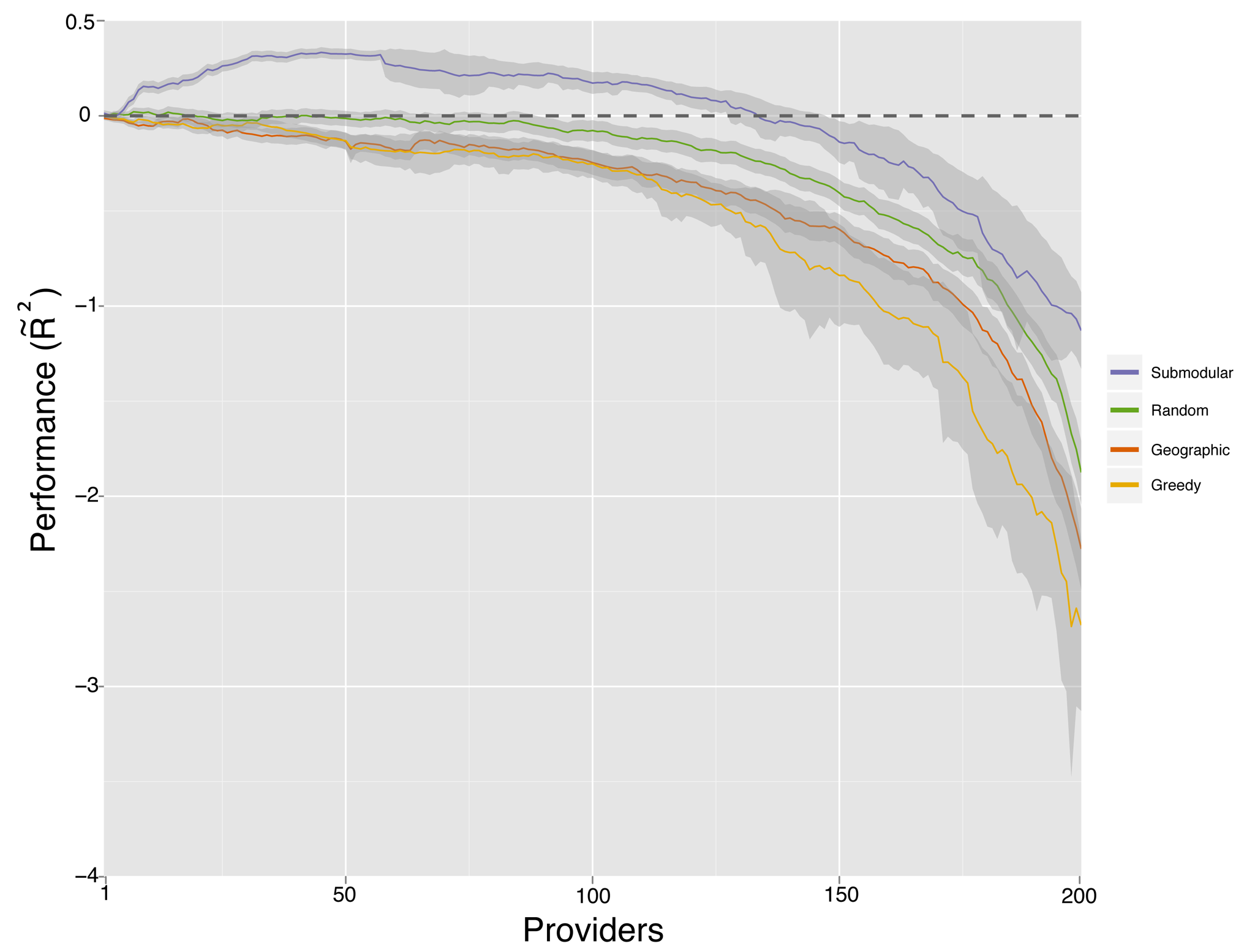

Supplement: Figure S4 — Out-of-Sample Model Validation - We used data from 2001–2007 to design ILINets and to fit multi-linear prediction functions, and then generated provider-report based forecasts of hospitalizations during 2008 (without using any data from 2008) and compared these predictions to actual 2008 hospitalization data (see text for details). The values reflect the predictive performance of the different ILINets. For each ILINet, we predicted 100 time series from simulated provider reports, each time drawing random deviates from the provider noise and reporting distributions, and then compared them to actual 2008 hospitalizations by calculating . Lines indicate the average and shaded regions indicate the middle of the distribution. Negative values indicate that the predicted hospitalization time series are more variable than the actual time series. The increasingly poor performance and uncertainty with additional providers is a result of over-fitting of the prediction model to data from the 2001–2007 training period. The submodular method is the only one to yield ILINets with a greater than zero. (TIF) [file pcbi.1002472.s004.tif]

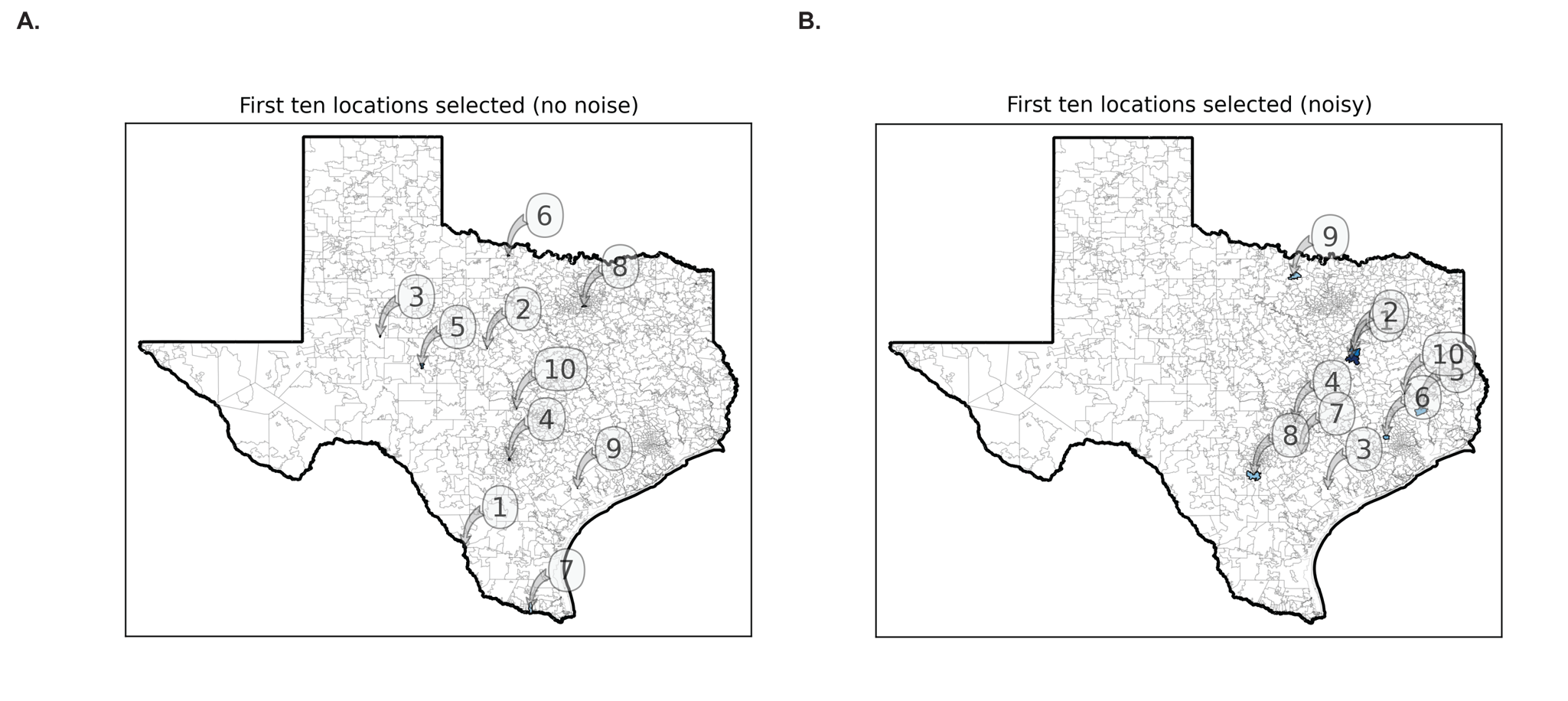

Supplement: Figure S5 — The importance of realistic reporting rates and noise - We compared the first ten providers selected by the submodular optimization method when providers either contained (a) perfect information and perfect reporting rates or (b) were subject to the patterns of imperfect and variable reporting exhibited by actual ILINet providers. When simulated providers had reporting probabilities and noise similar to actual providers the resulting network contained more geographic redundancy than one built from simulated providers with perfect information and reporting rates. All results presented in the manuscript were determined using simulated providers with patterns of imperfect and variable reporting derived from actual ILINet data. The stark difference highlights the importance of incorporating the characteristics of actual ILINet provider reporting. (TIF) [file pcbi.1002472.s005.tif]
