# Supplementary material for: Optimizing Provider Recruitment for Influenza Surveillance Networks
Source: PLoS Comput Biol. 2012 Apr 12;8(4):e1002472. doi: 10.1371/journal.pcbi.1002472 (PMC3325176; doi:10.1371/journal.pcbi.1002472)
Supplement: Text S1 — In text S1, we present the results of five supplementary analyses. 1) The importance of influenza-like hospitalizations in terms of total respiratory disease related hospitalizations and health care charges in Texas, 2) The details of actual ILINet provider reporting in Texas, the data described here were used to derive our provider reporting model, 3) The time-lagged, linear relationship between influenza-like hospitalizations, ILINet, and Google Flu Trends in Texas, 4) Additional model validation results, which support and confirm those presented in the main text, and 5) The importance of incorporating realistic provider reporting rates and noise illustrated by the dramatic difference in the results when perfect information and reporting is assumed. (PDF) [file pcbi.1002472.s006.pdf]

Running Title:

Supplement - Influenza Surveillance Network Design

Keywords

Public Health, Epidemiology, Influenza (Human), Surveillance

Supplement - Optimizing Provider Recruitment for Influenza Surveillance Networks

Samuel V. Scarpino[1,*], Nedialko B. Dimitrov[2], Lauren Ancel Meyers[1,3]
1 The University of Texas at Austin, Section of Integrative Biology, Austin, TX, USA
2 Naval Postgraduate School, Operations Research Department, Monterey, CA, USA
3 The Santa Fe Institute, Santa Fe, NM, USA
∗ E-mail: Corresponding scarpino@utexas.edu

1 ICD9 486, 487 and 488 associated hospitalizations in Texas

We quantified the importance of *influenza-like hospitalizations* [ICD9 486, 487 and 488] in Texas in three ways:

1. Depending on the time of year, *influenza-like hospitalizations* were associated with between 23 and 37 percent of all respiratory disease related hospitalizations, Figure S1

2. From 2002 - 2008, *influenza-like hospitalizations* were responsible for a total of 44.99 billion dollars in health care charges or an average of 470 million dollars per month, Figure S2.

To determine the total number of respiratory illness related hospital admissions, we used ICD9 460 - 519. To estimate the total cost of ICD9 486, 487, and 488 hospitalizations, we used the total charges field in the hospital admission records.

2 Reporting rates of actual ILINet providers in Texas

Participation in the Texas ILINet, as is the case with all ILINets, is voluntary. As a direct result, providers exhibit highly variable reporting rates. To model provider reporting, we first estimated four transition probabilities from the actual ILINet providers. These transition probabilities allowed us to model provider reporting as a Markov process where the probability that a provider reports on a given week is conditional upon their behavior in the previous week. This model was able to capture the two most striking features of ILINet provider reporting: 1.) low reporting rates on average and 2.) streaky reporting. The raw estimates of the four transition probabilities are presented in Figure S3a. Importantly, there were three main classes of providers in terms of how likely they are to report, how likely they are to continue reporting once they start, and how likely they are to resume reporting after a period of non-reporting. This feature can be seen in Figure S3b, where the lower left corner of the graph represents providers who rarely report and are unlikely to resume reporting after stoping, the lower right corner are those providers who report regularly but once they stop reporting are unlikely to resume, and the top right corner represents the best kind of provider, one who reports regularly and will likely resume reporting if they miss a week.

3 Influenza-like hospitalizations, ILINet, and Google Flu Trends in Texas

Implicit in our decision to use ILINet, Google Flu Trends, and hospital admission data is the assumption that they are correlated with each other. To investigate the relationship between these variables, we performed a series of time-lagged regressions. The results of these regressions can be seen in Table 1. The best fit relationship between Google Flu Trends and *influenza-like hospitalizations* occurs with a time lag of one week and has an R^2 of 0.74. For ILINet, the best fit model to *influenza-like hospitalizations* had a time lag of two weeks and had an R^2 of 0.66. Google Flu Trends and ILINet had a best fit model with a lag of zero weeks and an R^2 of 0.77, interestingly all lags greater than two weeks between Google Flu Trends and ILINet were non-significant. However, given that Google Flu Trends was designed to accurately represent the number of ILI cases, the results seem less surprising.

Table 1: R^2 between *influenza-like hospitalizations*, Google Flu Trends and ILINet in Texas

Dependent Variable	ILINet	Google Flu Trends
ICD9 (486,487,488), 0 week lag	0.56	0.62
Google Flu Trends, 0 week lag	0.77	—
ICD9 (486,487,488), 1 week lag	0.58	0.74
Google Flu Trends, 1 week lag	0.73	—
ICD9 (486,487,488), 2 week lag	0.66	0.72
Google Flu Trends, 2 week lag	0.43	—
ICD9 (486,487,488), 3 week lag	0.61	0.61
Google Flu Trends, 3 week lag	-0.04 (NS)	—
ICD9 (486,487,488), 4 week lag	0.49	0.47
Google Flu Trends, 4 week lag	-0.14 (NS)	—

4 Model Validation

To validate the results of our method, we simulated the prediction of future hospitalizations, using only historical data for creating the network and estimating the multilinear prediction function. We used hospitalization data from 2001-2007 to fit prediction functions for each network, and then used those prediction functions to forecast hospitalizations in 2008. In the main text, we evaluated our ILINets by comparing the forecasted hospitalizations to actual hospitalizations. Here, we present an alternative method for making this comparison.

Figure S4 depicts the prediction performance of four different ILINet designs. The horizontal axis gives the number of providers in the network. The vertical axis, gives a variance reduction measure similar to R^2, except that the linear regression coefficients are not determined from 2008 data. Specifically, let

$$\tilde{R}^2(\mathbf{G}^{2008}, S^{\text{train}}, \xi) = \frac{\text{Var}(\mathbf{G^{2008}}) - \text{Var}(\mathbf{G^{2008}} - \sum_{i \in S_{\text{train}}} \alpha_i^{\text{train}} \cdot P_i^{2008}(\xi))}{\text{Var}(\mathbf{G^{2008}})},$$

where G^{2008} is the hospitalization time series in 2008, S^{train} is the set of providers selected based on data from the training period (2001- 2007), ξ indicates a particular set of noise and reporting profiles for the providers, the coefficients α_i^{train} are estimated via multilinear regression of actual hospitalizations on

simulated provider data during 2001-2007 period, and $P_i^{2008}(\xi)$ are the mock provider reports in 2008. For each ILINet in Figure S4, we generated 100 random provider reports, each time choosing random provider noise profiles from the provider noise and reporting profile distributions, and used those reports to calculate 100 different $\tilde{R}^2$ values. Figure S4 shows the mean and middle 90% of the distribution of $\tilde{R}^2$ values for each ILINet-size combination. This calculation models a scenario where we first use historical data to create the ILINet, then use historical data to fit a prediction function, and finally use real-time provider reports to predict real-time hospitalizations. The difference between the $\tilde{R}^2$ and the typical R^2 is that the coefficients of the model (α_i^{train}) are determined in advance, based on historical data. The $\tilde{R}^2$ can be negative if the predicted values are more variable than the actual time series, which is impossible under a standard R^2 calculation following least squares regression.

The $\hat{R}^2$ depicted in Figure 7 differs from the $\tilde{R}^2$ calculated here in that $\hat{R}^2$ involves first calculating expected provider reports by averaging over multiple draws from the noise distributions (canceling out the noise), then predicting a hospitalization time series from those expected reports, and finally comparing the predicted time series to the actual time series from 2008; the $\tilde{R}^2$ involve predicting a separate hospitalization time series for each draw from the noise and reporting distributions, comparing it to the actual time series, and then averaging those comparisons over all draws.

Both the $\hat{R}^2$ (Figure 7) and $\tilde{R}^2$ (Figure S4) suggest that submodular optimization will outperform the other design methods. It is the only method to produce results with an $\tilde{R}^2$ significantly greater than zero. The degradation and the rough cutoff of 100 providers is a result of the input data used to design the network. From 2001 to 2007, there are 222 weeks of data. Any network of 222 providers would be able to produce a perfect R^2 for those points in-sample, simply because of linear independence in the provider reports. Thus, adding too many providers over-fits the prediction function in-sample, and produces poor results on the out-of-sample testing period.

5 Importance of realistic noise and reporting rates

We compared two networks constructed using the submodular optimization method (a) when simulated providers contained perfect information about *influenza-like hospitalizations* and perfect reporting rates and (b) when they had reporting rates and noise characteristic of real providers, Figure S5. When simulated providers had reporting probabilities and noise similar to actual providers the resulting network contained more geographic redundancy than one built from simulated providers with perfect information and reporting rates. All results presented in the manuscript were determined using simulated providers with patterns of imperfect and variable reporting derived from actual ILINet data.
